# Designing Green Plasticizers: Linear Alkyl Diol Dibenzoate Plasticizers and a Thermally Reversible Plasticizer

**DOI:** 10.3390/polym10060646

**Published:** 2018-06-09

**Authors:** Hanno C. Erythropel, Aurélie Börmann, Jim A. Nicell, Richard L. Leask, Milan Maric

**Affiliations:** 1 Department of Chemical Engineering, McGill University, 3610 University Street, Montréal, QC H3A 0C5, Canada; aurelie_b@gmx.de (A.B.); richard.leask@mcgill.ca (R.L.L.); 2 Department of Chemical and Environmental Engineering, Yale University, 10 Hillhouse Avenue, New Haven, CT 06511, USA; 3 Department of Civil Engineering & Applied Mechanics, McGill University, 817 Sherbrooke Street West, Montréal, QC H3A 0C3, Canada; jim.nicell@mcgill.ca

**Keywords:** green plasticizer, reversible heat-activated plasticizer, low toxicity profile, PVC, DEHP replacement, phthalates, glass transition temperature, tensile properties, torsional modulus, surface hardness

## Abstract

Several linear alkyl diol dibenzoate compounds, ranging from C3 to C6 in central diol length, were evaluated for their plasticizing effectiveness in blends with poly(vinyl chloride) (PVC). The results were compared to blends of PVC/di(2-ethylhexyl) phthalate (DEHP), the most commonly used commercial plasticizer. DEHP has come under scrutiny, due to its suspected endocrine-disrupting behaviour, and the proposed diol dibenzoates have previously been shown to have the potential to be green, safe candidates for DEHP replacement. The thermal and mechanical properties of PVC/dibenzoate blends were determined, and include glass transition temperature (T_g_), the elongation at break, maximum stress, apparent moduli, torsional modulus, and surface hardness. The C3, C5, and C6 dibenzoates performed as well as or better than DEHP, with the exception of torsional modulus, further supporting their use as green plasticizers. For blends with 1,4-butanediol dibenzoate, differential scanning calorimetry and torsional temperature sweeps suggested that the compound partly crystallizes within PVC blends over the course of two days, thereby losing the ability to effectively plasticize PVC. However, upon heating to temperatures above 60 °C, effective plasticization was again observed. 1,4-Butanediol dibenzoate is thereby a reversible heat-activated plasticizer or processing aid with excellent plasticizer properties at mildly elevated temperatures.

## 1. Introduction

**Figure 1:**
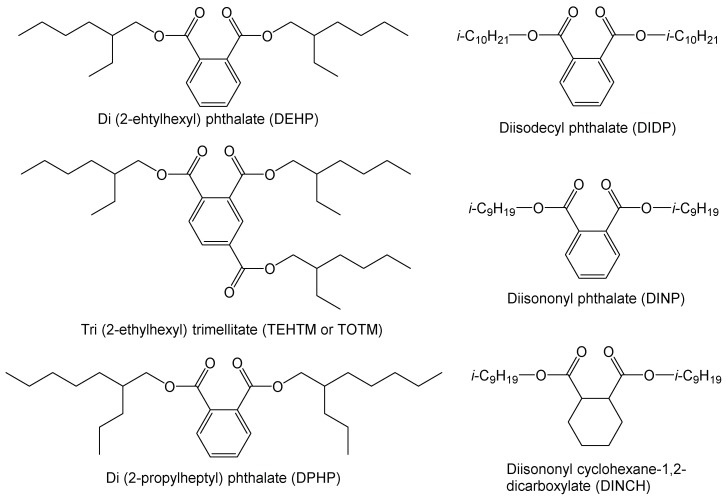
Chemical structures of important commercial plasticizers including four phthalates [di(2 ethylhexyl) phthalate (DEHP), di(2-propylheptyl) (DPHP), diisodecyl phthalate (DIDP), and diisononyl phthalate (DINP)], as well as two compounds that are structurally similar to phthalates: tris(2-ethylhexyl) trimellitate (TOTM) and diisononyl cycylohexane-1,2-dicarboxylate (DINCH).

Poly(vinyl chloride) (PVC) is one of the “big 6” polymers [1], with worldwide production capacities above 5 × 10^7^ tonnes per year [2]. The difficulty in processing pure PVC is remedied through the use of several additives, including plasticizers, heat stabilizers, internal and external lubricants, processing aids, and others [2]. However, these additives are not chemically bound to PVC, leading to the possibility of their leaching from the blend over time [3]. Plasticizers are of particular importance for PVC, to which they are commonly added in concentrations up to 30–40% by weight [4], in order to render the material soft and “plastic” by breaking up PVC chain–chain interactions [5]. For decades, the most important plasticizer class for PVC have been phthalates, which are diesters of ortho-phthalic acid and accounted for 70% of the world consumption of plasticizers in 2014 [6]. Within this class, di(2-ethylhexyl) phthalate (DEHP) is the most important common primary plasticizer for PVC (Figure 1) [7], yet recent data from 2016 by the European PVC industry suggests that several other phthalates and phthalate-resembling compounds have gained importance in Europe [8]. These compounds are diisononyl phthalate (DINP), diisodecyl phthalate (DIDP), di(2-propylheptyl) (DPHP), and trimellitate triesters, particularly tris(2-ethylhexyl) trimellitate (TOTM) (basically DEHP with an added ester function) and diisononyl cycylohexane-1,2-dicarboxylate (DINCH), which is hydrogenated DINP (see Figure 1) [8]. DEHP and several other phthalates have come under scrutiny, as they are considered to be ubiquitous global contaminants [3,9], and due to the suspicion that their breakdown products, such as mono(2-ethylhexyl) phthalate (MEHP), dysregulate hormonal activity [10,11,12,13,14]. It is for this reason that regulators in several major jurisdictions, including the United States, the European Union, Japan, and Canada have banned several phthalates, including DEHP, DINP, and DIDP, from children’s toys [3,15]. 

As a result, it is imperative that greener, safer plasticizers be designed and implemented commercially, especially given the predicted growth in plasticizer demand, especially in Asia [6]. Such plasticizers should be designed and produced with the whole life cycle in mind, and with a special focus on good plasticizing effectiveness, rapid biodegradability, and low to no toxicity, especially with respect to their impact on the reproductive system. The 12 Principles of Green Chemistry, as outlined by Anastas and Warner [16,17], serve as a good guideline for the task at hand; among others, these focus on the design stage in order to avoid future problematic compounds, on the production stage to avoid toxic starting materials, intermediates, auxiliaries, catalysts, and by-products, and on sustainable and renewable starting materials, rather than petroleum-based materials.

**Figure 2:**
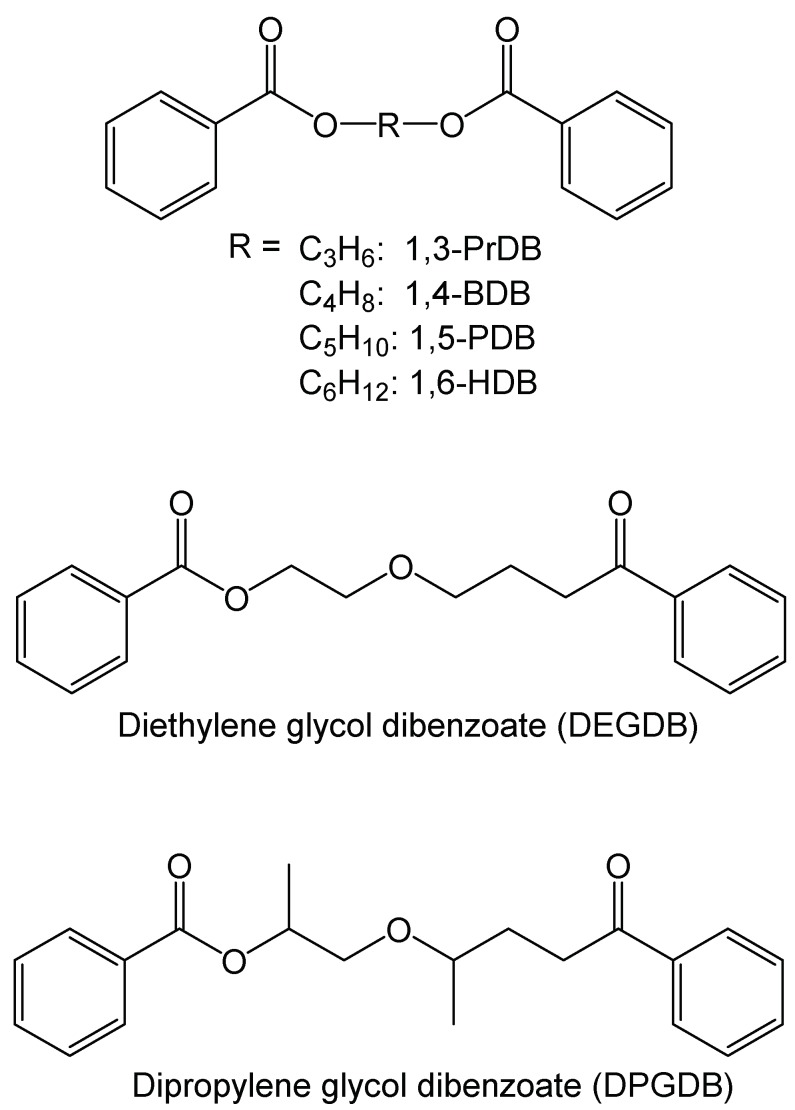
Chemical structures of the proposed diol dibenzoate plasticizers and two commercial dibenzoate plasticizers (diethylene glycol dibenzoate (DEGDB), dipropylene glycol dibenzoate (DPGDB)). Abbreviations: 1,3-PrDB is 1,3-propanediol dibenzoate; 1,4-BDB is 1,4-butanediol dibenzoate; 1,5-PDB is 1,5-pentanediol dibenzoate; and 1,6-HDB is 1,6-hexanediol dibenzoate.

While several commercial dibenzoate plasticizers, such as diethylene glycol dibenzoate (DEGDB) and dipropylene glycol dibenzoate (DPGDB; see Figure 2), are sold commercially [18,19], these compounds generally contain ether linkages in their central linker connecting the two benzoate moieties. Prior work has shown that the presence of these ether bonds leads to metabolites that are difficult to degrade microbially, and which are possibly more toxic than their parent compound [20]. In a previous study, 1,5-pentanediol dibenzoate (1,5-PDB, see Figure 2) has been proposed and tested as a PVC plasticizer, since the only difference between 1,5-PDB and DEGDB lies within the diol linker part: the oxygen atom of the ether bond in DEGDB is replaced with a carbon atom in 1,5-PDB. The previous study demonstrated that 1,5-PDB was as effective in plasticizing PVC as DEGDB and DEHP. However, 1,5-PDB had the advantage in that the modified linker resulted in more rapid biodegradation by common soil bacteria, in comparison to DEGDB [21]. This was expected, since it is known that unbranched alkanes are more readily biodegraded than ethers [22].

In addition to 1,5-PDB, the related compounds 1,3-propanediol dibenzoate (1,3-PrDB), 1,4-butanediol dibenzoate (1,4-BDB), and 1,6-hexanediol dibenzoate (1,6-HDB) (see Figure 2) have been shown to be biodegraded quickly by common soil bacteria without significant metabolite accumulation [23,24]. Furthermore, all four diol dibenzoates showed low toxicity profiles to the bacteria utilized in the MicroTox^®^ assay (except 1,3-PrDB) [25] and low toxicity profiles in several mammalian cell line in-vitro assays [26,27]. Furthermore, 1,4-BDB was found to not significant alter adult male reproductive function in an in-vivo rat study [28,29]. 

These are all positive indications of the potential of these alternative compounds as potential plasticizers, but it remains to be seen whether their plasticizing qualities approach those of phthalates. As such, this study aimed at documenting the plasticizer effectiveness of all four mentioned diol dibenzoates and comparing the results to similar measurements of the commercial plasticizer DEHP, in order to establish whether or not these compounds are suitable green plasticizer candidates.

## 2. Methods and Materials

### 2.1. Synthesis

The four diol dibenzoate compounds 1,3-PrDB, 1,4-BDB, 1,5-PDB, and 1,6-HDB (Figure 2) were synthesized as described previously [21,24,30]. Briefly, their corresponding diols—1,3-propanediol, 1,4-butanediol, 1,5-pentanediol, and 1,6-hexanediol (all > 97% pure, Sigma-Aldrich, Oakville, ON, Canada)—were refluxed with benzoyl chloride (99%, Sigma-Aldrich, Oakville, ON, Canada) for several hours, then neutralized and purified by re-crystallization from heptane. NMR spectra were recorded to confirm the chemical structure. These spectra are shown in the Supplemental Material. 

To ensure the compounds were suitable for processing, the melting points (m.p.) were determined for all four compounds, both visually by using a melting point apparatus (Gallenkamp, Loughborough, UK) and by differential scanning calorimetry (DSC). The recorded melting points are listed in Table 1.

### 2.2. Extrusion of Poly(Vinyl Chloride)/Plasticizer Blends

Unplasticized PVC (UPVC; K50) was obtained from Solvay-Benvic (Chevigny Saint Sauveur, France). As described previously [15,21], an intermeshing twin-screw extruder with conical screws of 5/14 mm diameter and 109.5 mm length (Haake Minilab, Thermo Fisher Scientific, Burlington, ON, Canada) was used to blend UPVC with the candidate plasticizers. Extrusion to 40 parts per hundred rubber (phr), corresponding to approximately 29 wt %, was carried out in two steps: first, by incorporation of 20 phr of plasticizer candidates, along with 4 phr epoxidized soybean oil as heat stabilizer (Chemtura, Elmira, ON, Canada) and 5 phr stearic acid as lubricant (Thermo Fisher Scientific, Burlington, ON, Canada) at 130 °C; and subsequently, by extrusion of the 20 phr blend to 40 phr via the addition of more plasticizer candidate at 120 °C. The extruder was operated in batch mode with a batch size of 3 g, and approximately 5 batches per step were prepared. For each batch, the screw speed was set to 30 rpm for 5 min, and raised to 60 rpm for 2 min, after which the next batch was added. All material was passed through the extruder twice per step to ensure homogeneity.

### 2.3. Mechanical Test Bar Production

To produce (1) tensile testing bars, as per ASTM D-638 [31]; (2) rectangular test bars for dynamic mechanical thermal analysis DMTA analysis, as per DMTA D-4065 [32], and (3) surface hardness testing, as per ASTM E-2546 [33], a hot press (Carver manual hydraulic press with Watlow temperature controllers, Wabash, IN) was used to mold the finely cut blends into the desired shape at 170 °C for 40 min. Subsequently, the hot press was cooled down to approximately 50 °C using tap water flowing through the internal piping, and the produced tensile testing or rectangular bars were removed carefully. The test bar dimensions used were those required by the ASTM standards, as follows: (1) 1.5 mm thickness, 3.25 mm width of narrow section (W), 15.5 mm length of narrow section (L), 32.5 mm distance between grips (D), 63.5 mm overall length (LO), and 10 mm width overall (WO); and (2) 1.5 mm thickness, 10 mm width, and 50 mm length. All test bars were conditioned for 48 h in a desiccator at room temperature before further testing.

### 2.4. Differential Scanning Calorimetry (DSC)

For an assessment of the thermal properties of PVC/plasticizer blends, both DSC and temperature-modulated DSC (MDSC) were performed (TA Instruments Q2000, Grimsby, ON, Canada) using a standard DSC pan (TA Instruments Model No. 070221). The pan was filled with several thin slices of extruded PVC/plasticizer blend and the top crimped on, to yield a final sample weight of roughly 10 mg (DSC) or 2 mg (MDSC), respectively, which was recorded using a balance (Sartorius CP225D, Göttingen, Germany). Using the autosampler, the sample and a reference pan were loaded into the machine, and the following temperature regime was applied: for DSC, the sample was quenched at −90 °C for 5 min, followed by heating to 100 °C at a heating rate of 10 °C/min. This sequence was repeated a second time, and unless otherwise indicated, the second heating cycle was used to extract thermal properties of the material. For MDSC, a similar method was used, except that the heating rate was set to 2 °C/min and superimposed with a sinusoidal modulation of 1.27 °C with a period of 60 s. Generally, glass transition temperatures were determined using the half-height method, using the analysis software “TA Universal Analysis” (TA Instruments).

Regular DSC was also carried out to determine the melting points (T_m_) of the pure alkyl diol dibenzoates, loading a sample size between 2 and 5 mg in a standard DSC pan (TA Instruments Model No. 070221), and running two consecutive scans from −20 °C to 100 °C at a heating rate of 10 °C/min. T_m_ was determined per cycle using the “onset point” function integrated into “TA Universal Analysis”.

### 2.5. Dynamic Mechanical Thermal Analysis (DMTA)

For DMTA analysis, a rheometer with convection oven, fixed bottom clamp, and moveable top clamp was used (Anton Paar MCR302 with SRF12 fixtures and CTD450 convection oven, Saint-Laurent, QC, Canada), and run under nitrogen purge to avoid sample oxidation. The exact dimensions of the middle section of the mechanical test bars were recorded (Electronic Outside Micrometer, Fowler Precision, Auburndale, MA, United States), and the sample was attached appropriately. DMTA was run in torsion mode, and frequency sweeps were carried out from 1 to 100 rad/s (0.16 Hz to 16 Hz) at 25 °C, strain-controlled with amplitude γ = 0.1% and normal force −1 N. Temperature sweeps were carried out from 30 to 100 °C at 1 Hz with amplitude γ = 0.01% and normal force −0.5 N. The software RheoPlus V3.61 (Anton Paar, Saint-Laurent, QC, Canada) was used to extract storage moduli G’, loss moduli G”, and tan δ values.

### 2.6. Tensile Testing

For tensile testing, a Shimadzu EZ Test tensile tester (Burlington, ON, Canada) was used with a 500-N load cell as per ASTM D-638 [31], at room temperature. The exact dimensions of the middle section of the mechanical test bars were measured (Electronic Outside Micrometer, Fowler Precision, Auburndale, MA, United States), the sample was attached appropriately, and the experiment was carried out at a strain rate of 5 mm/min. Strain at break (in % elongation (EL)) and maximum observed stress (in MPa) were extracted from the recorded stress–strain curves and the apparent moduli at 10% EL, 25% EL, 50% EL, and 75% EL (in MPa) were calculated by using the derivative of a polynomial fit to the experimental data (generally sixth order, with an *R*^2^ of at least 0.98).

### 2.7. Surface Hardness by Nano-Indentation

To determine surface hardness, a nano-indenter (Nanovea PB1000 with Nano module, Irvine, CA, United States) with a stainless-steel ball tip of d = 1 mm was used, at room temperature. Three measurements per mechanical test bar were performed, each in a different third of its length. After zeroing the tip to the sample surface, it was forced into the sample at 30 mN/min up to a total load of 20 mN, and subsequently removed at the same rate of 30 mM/min while recording indentation depth. A Matlab^®^ program was used to calculate the surface hardness according to ASTM E-2546 [33]. Briefly, the calculation was based on a stiffness term derived from the slope of the first third of the unloading curve and the estimated contact area and indentation depth. The results were expressed in units of MPa.

### 2.8. Microscopy

Qualitative microscopy images at 40× magnification of PVC blends with 1,4-BDB and DEHP were recorded with a Lumenera Infinity 1 camera (Ottawa, ON, Canada) attached to a Leitz Diaplan microscope (Wetzlar, Germany) with a polarized light filter.

### 2.9. Statistical Analysis

Statistical analyses were carried out using GraphPad Prism 7.01 software (La Jolla, CA, United States). Unless specified otherwise, one-way ANOVA tests with Bonferroni post-tests were performed. Reported *p* values stemmed from the post-test. Detailed statistical analysis results are provided in Table S3 in the Supporting Material.

## 3. Results

Several plasticizer properties of the four dibenzoates with varying central linker length were evaluated when blended with UPVC, and the results were compared to blends at equal phr of commercial DEHP, shown in Table 2. Plasticized PVC blends commonly contain high concentrations of plasticizer ranging from 30 to 60 phr or higher [34]; a concentration of 40 phr was chosen for this study, corresponding to approximately 29 wt %, which is well within the commercial range. Values for 1,4-BDB are not shown, since blends with this compound seemed to harden out over time (see below).

### 3.1. Glass Transition Temperature (T_g_)

The T_g_ was measured by DSC as an indicator of plasticizer compatibility and effectiveness. All values were in close vicinity to one another, (−5.4 °C to 2.9 °C), yet the T_g_ values of blends with 1,3-PrDB and 1,4-BDB were significantly higher than those of blends prepared with DEHP (Bonferroni post-test; *p* = 0.002 and *p* = 0.003, respectively). The T_g_ values of blends prepared with both 1,5-PDB and 1,6-HDB were not statistically different from those prepared with DEHP (*p* = 0.601 and *p* > 0.999, respectively).

**Figure 3:**
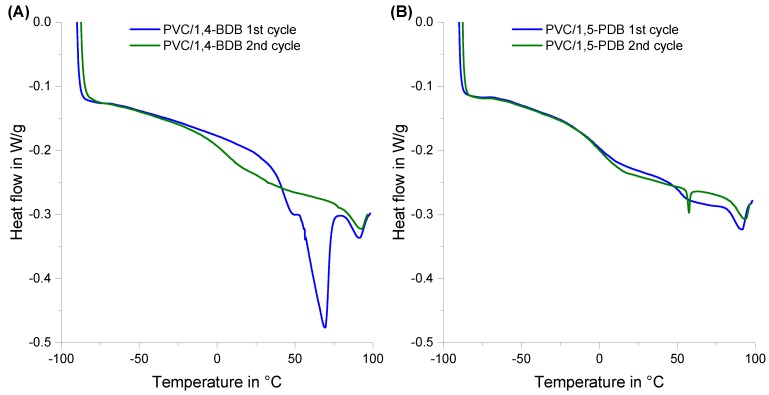
DSC curves for the first (blue) and second (green) heating cycles of a 40 phr blend of (**A**) PVC/1,4-BDB and (**B**) PVC/1,5-HDDB.

An anomaly was noticed for 1,4-BDB blends; that is, the extruded blends were soft and flexible even after cooling, yet when the samples were prepared for DSC analysis several days later, the blend was very hard and rigid. This behavior is mirrored in the DSC curves shown in Figure 3A, where in the first heating cycle, no T_g_ was apparent, but a large non-reversible heat flow started at around 50 °C, indicative of a non-reversible transition, such as a melting process. In the second heating cycle, a clear T_g_ transition appeared in the same range as for blends with the other dibenzoate candidates (e.g., for 1,5-PDB shown in Figure 3B), yet no non-reversible transition was observed in the second heating cycle for a blend containing 1,4-BDB.

### 3.2. Dynamic Mechanical Thermal Analysis (DMTA) in Torsion Mode

DMTA torsion tests were carried out as (a) frequency sweeps from 1 to 100 rad/s (0.16 to 16 Hz) at 25 °C and (b) as temperature sweeps from 30 to 100 °C, at a constant frequency of 1 Hz, in order to compare the torsional modulus parameters G’ (storage modulus) and G” (loss modulus) of blends of the dibenzoate candidates in PVC to blends containing DEHP. The G’ and G” for 1 Hz at 25 °C are reported in Table 2 and demonstrate a lower torsional modulus of 1,5-PDB compared to DEHP. Blends of 1,6-HDB and 1,3-PrDB were stiffer in torsional mode than DEHP, as evidenced by higher moduli, but were of the same order of magnitude.

**Figure 4:**
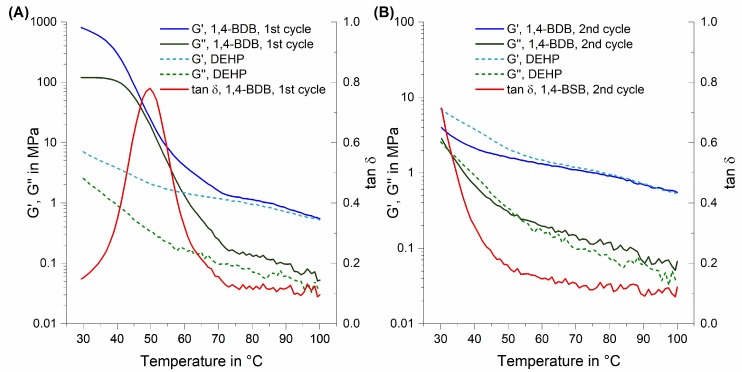
Log plot of torsional DMTA temperature sweep curves: G’ (blue) and G” (green) for 40 phr blends of PVC/1,4-BDB and PVC/DEHP, and tan δ (red) for PVC/1,4-BDB in temperature sweep mode. (**A**) The first heating cycle; and (**B**) the second heating cycle, started within 1 h of the first.

The test bars containing 1,4-BDB felt very rigid compared to all other test bars after the standard 48 h of desiccation, and the temperature sweep was carried out twice in direct succession, similar to the T_g_ measurements. As shown in Figure 4, a similar pattern was observed; that is, the first temperature sweep showed a high torsional modulus until the appearance of a large tan δ peak just below 50 °C, indicative of a phase transition. After this transition, the torsional modulus was in the vicinity of samples with DEHP (log plot in Figure 4A). A second temperature sweep recorded within an hour of the first temperature sweep lacks this peak in tan δ, and recorded G’ values were initially lower for 1,4-BDB blends than for those containing DEHP, but close to blends with DEHP above T = 60°C (log plot in Figure 4B).

### 3.3. Tensile Testing

**Figure 5:**
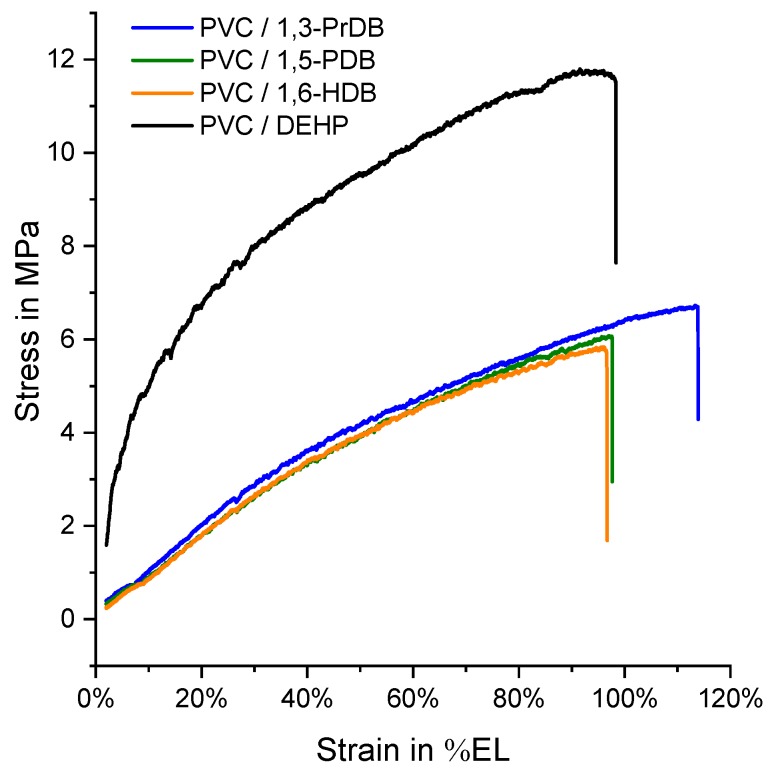
Examples of stress-strain curves of 40 phr blends of PVC with 1,3-PrDB, 1,5-PDB, 1,6-HDB, and DEHP. Results for 1,4-BDB are shown in Figure S1 and Table S2. Apparent moduli at 25% EL are listed in Table 2 and at 10% EL, 50% EL, and 75% EL in Table S1.

Tensile testing was carried out at room temperature to obtain stress–strain curves for PVC blended individually with each of four diol dibenzoate compounds, or with DEHP. Examples of one curve per plasticizer candidate (except 1,4-BDB) are shown in Figure 5, and elongation at break in % EL, maximum recorded stress in MPa, and apparent modulus at 25% EL in MPa were determined for each compound, as show in Table 2. The apparent modulus at 25% EL, corresponding to the slope of the curve at 25% EL, is reported rather than Young’s modulus, due to the non-linearity of the stress–strain curves (Figure 5), as reported previously for other classes of proposed green plasticizers [15]. The apparent moduli at 10% EL, 50% EL, and 75% EL were also calculated, and are reported in Table S1. As above, blends containing 1,4-BDB were stiff, and an example for a stress–strain curve for a blend containing 1,4-BDB is shown Figure S1.

Only blends with 1,3-PrDB exhibited statistically significantly higher elongation at break versus blends of all the other compounds [i.e., *p* = 0.002 (versus 1,5-PDB), *p* = 0.007 (versus 1,6-HDB), *p* = 0.003 (versus DEHP)]. In terms of maximum observed stress, statistically significantly different values were observed for all blends, with the exception of those containing 1,5-PDB and 1,6-HDB (*p* = 0.672; see also Figure 5). Furthermore, no statistically relevant difference existed between the calculated apparent moduli at 25% EL for the three dibenzoate candidates (Table 2), however, all three dibenzoate candidates (1,3-PrDB, 1,5-PDB, and 1,6-HDB) blends had significantly lower apparent moduli at 25% EL than DEHP (all *p* < 0.0001).Similar results were obtained for the apparent modulus at 10% EL (Table S1). Once higher strains are reached, the apparent moduli at both 50% EL and 75% EL do reveal statistical differences between the dibenzoate compounds (Table S1).

### 3.4. Surface Hardness by Nano-Indentation

**Figure 6:**
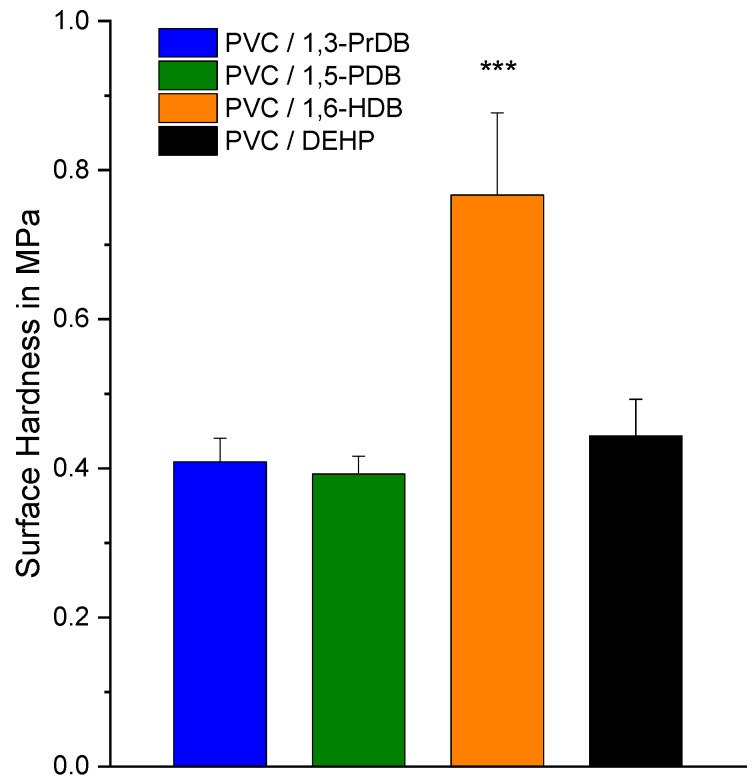
Surface hardness of 40 phr blends of PVC with 1,3-PrDB, 1,5-PDB, 1,6-HDB, and DEHP, as determined by nano-indentation. Standard deviation is shown with *n* = 3 for each of the diol dibenzoate compounds and *n* = 5 for DEHP. Results for 1,6-HDB were statistically significantly different than those of blends of all other compounds (*p* < 0.001).

The surface hardness of blends containing each of the plasticizer candidates was measured by nano-indentation at room temperature. The results indicate no statistically significant differences between surface hardness of blends containing 1,3-PrDB, 1,5-PDB, and DEHP (all *p* > 0.999; Figure 6), yet the surface hardness for the three compounds were statistically different from 1,6-HDB (all *p* < 0.001). As expected, 1,4-BDB exhibited higher surface hardness than all other blends (Table S2).

### 3.5. Microscopy

**Figure 7:**
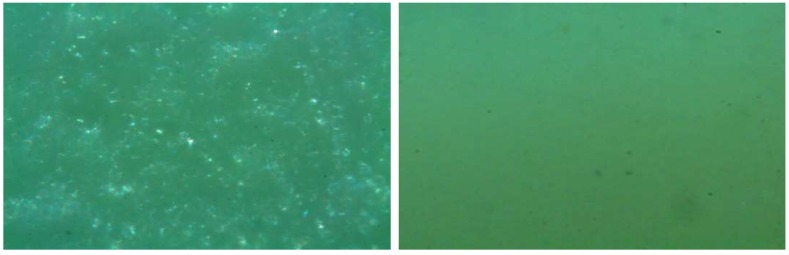
Microscope images (40×) with a polarized light filter of PVC blends containing 40 phr of 1,4-BDB (**left**) and DEHP (**right**), taken 48 h after test bar production.

To further investigate the effect of the hardening of the PVC blends with 1,4-BDB, a surplus DMTA test bar was placed under a microscope at a magnification of 40× (48-h post-pressing), and a polarized light filter was used to image the surface (Figure 7A). For comparison, a blend containing DEHP was analyzed in a similar manner at a similar time post-pressing (Figure 7B). The images show that the surface of the 1,4-BDB-containing blend was covered in what appears to be a crystalline material that reflected the light shone onto the sample, whereas such reflection was not observed for the PVC blend containing DEHP.

## 4. Discussion

This study demonstrates that three diol dibenzoate compounds with alkyl central linker lengths of C3, C5, and C6 (Figure 2) are as effective, or more effective than the commercial plasticizer DEHP, as demonstrated by glass transition temperature, torsional modulus, tensile strength, and surface hardness measurements. Further, the proposed diol dibenzoate compounds could be good green candidates to replace phthalates, given previous work that identified their rapid biodegradation by soil bacteria [21,24,30]. Several toxicological in-vitro studies have also suggested a low hazard associated with these compounds [26,27,28,29,35], yet a more thorough toxicological analysis would likely be needed to confirm these findings.

The C4 compound 1,4-butanediol dibenzoate (1,4-BDB) showed interesting behavior, in that the plasticizer seemingly loses its plasticization effectiveness over time, likely due to plasticizer crystallization within the blend. This behavior could be exploited for use in PVC processing as a reversibly heat-activated plasticizer, to shape the material at temperatures above 50 °C and subsequent material hardening at room temperature or below.

### 4.1. Dibenzoate Plasticizer Effectiveness in Poly(Vinyl Chloride) 

The effectiveness of the tested alkyl diol dibenzoate compounds was assessed as a combination of the various reported material properties. Of the four compounds tested in this study, 1,3-PrDB, 1,5-PDB, and 1,6-HDB (Figure 2) proved to be as effective in plasticizing PVC as the widely- and commercially-used plasticizer DEHP in blends at 40 phr. In particular, as seen in Table 2, 1,5-PDB showed similar or better plasticizing effectiveness in PVC compared to DEHP for all tested material properties, i.e., T_g_ reduction, elongation at break, maximum tensile stress, apparent modulus, torsional modulus (G’ and G”), and surface hardness. These results confirm and expand the scope of reported material properties [21] and rheological behavior [36] of PVC plasticized with 1,5-PDB, and lend further support for the excellent plasticizer properties of 1,5-PDB. As outlined by Firlotte *et al.*, 1,5-PDB resembles the commercial diethylene glycol dibenzoate (DEGDB), except that the oxygen atom of the ether linkage in the central part of DEGDB is replaced with a carbon atom (Figure 2) [21]. It is well established that ethers are much harder to degrade by microbes in the environment, in comparison to unbranched alkanes, such as is present in 1,5-PDB [22]. This explains the reported quicker biodegradation rates of 1,5-PDB compared to DEGDB [21].

The only structural difference between 1,3-PrDB, 1,6-HDB, and 1,5-PDB is the length of the central diol linker, yet the reported results suggest that the linker length is only marginally important; notably, blends containing 1,3-PrDB, 1,5-PDB, and 1,6-HDB produced stress–strain curves of almost identical shapes (Figure 5), resulting in similar apparent moduli (Table 2 and Table S2). Additionally, T_g_ reduction and surface hardness results also showed no significant differences between the three compounds in PVC blends (except 1,6-HDB). The discrepancy in surface hardness observed between 1,3-PrDB and 1,5-PDB compared with 1,6-HDB could be due to a surface specific effect, such as reduced 1,6-HDB surface mobility. Differences between bulk volume properties (tensile testing) and surface properties (surface hardness) in polymers are not uncommon and have been described previously—for example, for polypropylene (PP) [37]. Differences between the three diol dibenzoate compounds were further observed for the torsional modulus (Table 2). When comparing the results of 1,3-PrDB and 1,6-HDB to those of the commercial plasticizer DEHP, the results show that these compounds are either as effective or more effective in terms of all tested material properties, except for the torsional moduli (Table 2).

### 4.2. The Special Case of 1,4-Butanediol Dibenzoate (1,4-BDB)

Blends containing 1,4-BDB blends exhibited an anomaly; that is, even though they were soft after processing that involved heating the blends, such as extrusion and hot pressing, they felt hard and brittle to the touch after they were allowed to sit for 24 to 48 h. This observation is consistent with experimental results in which the PVC/1,4-BDB blends went through two heating cycles, such as DSC for T_g_ determination (Figure 3) or DMTA (Figure 4), where the second heating cycle showed similar behavior to the blends containing the other three diol dibenzoate candidates, but the first heating cycle did not. Data from both DSC and DMTA runs suggest that in the first heating cycle, a melting process occurs. One reasonable explanation for this could be that 1,4-BDB, a crystalline material in pure form, partially crystallized within the blend, thereby losing the ability to effectively plasticize PVC. As outlined in Table 1, the melting point of pure 1,4-BDB is 80–81 °C, yet the phase transitions observed in the first heating cycle of PVC containing 1,4-BDB were approximately 50–65 °C (DSC, much broader peak than for pure 1,4-BDB) and 50 °C (DMTA). This discrepancy can be explained by noting that the blend constitutes a mixture, and it is commonly known that mixtures lead to melting point depression of individual constituents [38]. The microscopic images shown in Figure 7 further suggest some sort of crystallization process; that is, small inclusions in the blend were observed on the surface of a blend containing 1,4-BDB 48 h after processing, when imaged with polarized light; this was not observed for a DEHP-plasticized blend after similar treatment, an unsurprising result since neither DEHP nor PVC are crystalline in pure form. The tendency of 1,4-BDB to crystallize rapidly was further observed during the visual melting point determination experiment, during which 1,4-BDB was found to form crystals very rapidly upon cooling following its removal from the instrument, whereas 1,3-PrDB, 1,5-PDB, and 1,6-HDB remained liquid even after cooling back to room temperature. One possible explanation for why only 1,4-BDB displays such rapid crystallization behavior could be favorable stacking of the two benzyl groups, facilitated by ideal spacing created by the 1,4-butanediol spacer [39,40,41].

While the behavior described above renders 1,4-BDB unsuitable as a simple drop-in replacement for DEHP due to the observed hardening of the blend, it could be used as a thermally reversible temperature-activated plasticizer or processing aid, since it exhibits good plasticizing effectiveness in PVC blends at temperatures above approximately 50 °C. For example, 1,4-BDB-plasticized PVC could be heated to the mild temperature of 50–60 °C, and then molded or otherwise formed into a desired shape, which it will retain once the material has hardened (for example, at room temperature). This process would also be reversible, by simply heating the material again. It should be noted that another positive aspect of 1,4-BDB is that the central diol linker can be produced renewably by microbial fermentation, or by reducing microbially-produced succinic acid [42], thereby lending to its character as a green plasticizer.

### 4.3. Diol Dibenzoate Safety

For any plasticizer to truly qualify as a suitable replacement for problematic commercial plasticizers such as DEHP, many aspects beyond plasticizer performance need to be addressed, and the principles of green chemistry can provide a good framework for such an assessment [16,17,43]. The discussed diol dibenzoate compounds showed good plasticizing effectiveness in PVC, andcoupled with the low toxicity profiles of the compounds in bacteria (except 1,3-PrDB) [25], mammalian cell in-vitro assays [26,27], and an in-vivo study of 1,4-BDB with a focus on reproductive toxicity [28,29], these compounds appear to be good green plasticizer candidates. However, further toxicity testing to meet regulatory needs is required. It should also be noted that the diol linker 1,4-butanediol is a precursor to the sedative γ-hydroxy butyric acid [44]. Therefore, a careful study assessing the possible breakdown of 1,-BDB to yield 1,4-butanediol is needed.

## 5. Conclusions

This work further outlines the suitability of several diol dibenzoate compounds, ranging in central diol linker length from C3 to C6, to effectively plasticize PVC. Three of the four compounds were as effective, or indeed, more effective than commercially-available DEHP in all but one tested material property. In combination with previous studies that showed rapid environmental degradability [21,24,30] and a low toxicity profile [26,27,28,29,35], 1,5-pentanediol dibenzoate in particular stands out as a good candidate green plasticizer to replace DEHP, in addition to 1,3-propanediol dibenzoate and 1,6-hexanediol dibenzoate. Further, while 1,4-butanediol dibenzoate is not a good candidate for a drop-in replacement of DEHP, due to its observed material hardening, this study suggests its use as a reversible heat-activated plasticizer or processing aid, with excellent plasticizer properties at mildly elevated temperatures.

## Figures and Tables

**Table 1:** Melting points (m.p.) of the four dibenzoate compounds, as established by differential scanning calorimetry (DSC) and by visual observation using a Gallenkamp melting point apparatus.

Dibenzoate Compound	Melting Point (°C)	
	By DSC	By Gallenkamp
1,3-propanediol DB	57–59	55–57
1,4-butanediol DB	80–81	78–80
1,5-pentanediol DB	25	20 < m.p. < 37 (body T)
1,6-hexanediol DB	54–55	54–55

**Table 2:** Glass transition temperature (T_g_), storage moduli (G’), and loss moduli (G”), as determined by dynamic mechanical thermal analysis (DMTA) in torsion mode, elongation at break, maximum observed stress, and apparent modulus at 25% EL, as determined by tensile testing, as well as surface hardness as determined by nano-indentation of 40 phr blends of PVC with the candidate plasticizers.

Candidate Plasticizer	T_g_ (°C) by DSC (*n* = 3)	DMTA Torsion (at 25°C) (*n* = 1)		Tensile Testing (at 20°C) (*n* = 4)			Hardness (at 20°C) (*n* = 3)
		G’ at 1 Hz (MPa)	G” at 1 Hz (MPa)	Elongation at Break (%EL)	Max. Stress (MPa)	Apparent Mod. at 25%EL (MPa)	Surface Hardness (MPa)
1,3-propanediol DB	2.9 ± 1.1	53.4	20.3	119 ± 7	6.8 ± 0.3	8.3 ± 0.3	0.41 ± 0.03
1,4-butanediol DB	n.o./2.5 ± 3.1 ^a^	-	-	-	-	-	-
1,5-pentanediol DB	−2.3 ± 0.5	7.07	4.68	95 ± 7 ^b^	6.1 ± 0.3 ^b^	8.5 ± 0.3 ^b^	0.39 ± 0.02
1,6-hexanediol DB	−3.5 ± 2.3	26.6	9.26	98 ± 10	5.8 ± 0.2	7.9 ± 0.5	0.77 ± 0.11
DEHP	−5.4 ± 0.5	10.5	4.27	96 ± 4	11.8 ± 0.1	12.1 ± 0.6	0.44 ± 0.05 ^b^

^a^ n.o. is not observed; two separate results recorded for two heating cycles, separated by the slash: no T_g_ observed on first heating cycle, second value represents data obtained in second heating cycle (see Figure 3A); ^b^ *n* = 5.

## Supplementary Materials

The following are available online at http://www.mdpi.com/2073-4360/10/6/646/s1, The supporting information contains the ^1^H-NMR data of the four candidate plasticizer diol dibenzoates, as well as Figure S1: Example of a stress–strain curve of a 40 phr blend of PVC/1,4-BDB; Table S1: Apparent moduli at 10% EL, 25%EL, 50% EL, and 75% EL for three diol dibenzoate candidates and DEHP; Table S2: Material property data collected for 40 phr PVC/1,4-BDB blends at room temperature, at least 48 h after processing; and Table S3: Statistical analysis results.
