# Supplementary material for: Designing Green Plasticizers: Linear Alkyl Diol Dibenzoate Plasticizers and a Thermally Reversible Plasticizer
Source: Polymers (Basel). 2018 Jun 9;10(6):646. doi: 10.3390/polym10060646 (PMC6404088; doi:10.3390/polym10060646)
Supplement: Supplementary file 1 [file polymers-10-00646-s001.pdf]

polymers

Supplemental Material

Designing Green Plasticizers: Linear Alkyl Diol Dibenzoate Plasticizers and a Thermally Reversible Plasticizer

Hanno C. Erythropel [1,2,*], Aurélie Börmann [1], Jim A. Nicell [3], Richard L. Leask [1] and Milan Maric [1,*]

1 Department of Chemical Engineering, McGill University, 3610 University Street, Montréal, QC H3A 0C5, Canada; aurelie_b@gmx.de (A.B.); richard.leask@mcgill.ca (R.L.L.)
2 Department of Chemical and Environmental Engineering, Yale University, 10 Hillhouse Avenue, New Haven, CT 06511, USA
3 Department of Civil Engineering & Applied Mechanics, McGill University, 817 Sherbrooke Street West, Montréal, QC H3A 0C3, Canada; jim.nicell@mcgill.ca
* Correspondence: hanno.erythropel@mail.mcgill.ca (H.C.E.); milan.maric@mcgill.ca (M.M.)

^{1}H-Nuclear Magnetic Resonance (NMR) spectroscopy

^{1}H-NMR spectroscopy was carried out on a Varian Mercury-300 (Palo Alto, CA, United States) with an average of eight repetitions. The solvent used for all measurements was deuterated chloroform ($CDCl_3$), with tetramethylsilane (TMS) as internal standard. The chemical shifts δ are shown in parts per million (ppm).

1,3-Propanediol dibenzoate: 1H-NMR (300.1 MHz in CDCl3): δ (ppm) = 2.25 [m, 2H, CH2CH2CH2], 4.50 [t, 4H, COOCH2CH2], 7.45 [m, 4H, Ar-H], 7.55 [m, 2H, Ar-H], 8.05 [m, 4H, Ar-H].

1,4-Butanediol dibenzoate: 1H-NMR (300.1 MHz in CDCl3): δ (ppm) = 2.0 [m, 4H, CH2(CH2)2CH2], 4.4 [t, 4H, COOCH2CH2], 7.45 [m, 4H, Ar-H], 7.55 [m, 2H, Ar-H], 8.05 [m, 4H, Ar-H].

1,5-Pentanediol dibenzoate: 1H-NMR (300.1 MHz in CDCl3): δ (ppm) = 1.65 [m, 2H, O(CH2)2CH2], 1.9 [m, 4H, OCH2CH2CH2], 4.35 [t, 4H, COOCH2CH2], 7.45 [m, 4H, Ar-H], 7.55 [m, 2H, Ar-H], 8.05 [m, 4H, Ar-H].

1,6-Hexanediol dibenzoate: 1H-NMR (300.1 MHz in CDCl3): δ (ppm) = 1.55 [m, 4H, O(CH2)2(CH2)2(CH2)2O], 1.85 [m, 4H, OCH2CH2CH2], 4.35 [t, 4H, COOCH2CH2], 7.45 [m, 4H, Ar-H], 7.55 [m, 2H, Ar-H], 8.05 [m, 4H, Ar-H].

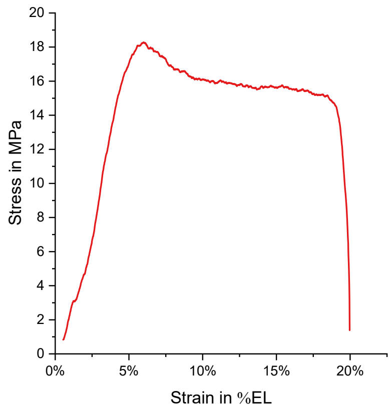

Figure S1. Example of a stress–strain curve of a 40 phr blend of PVC/1,4-BDB.

Table S1. Apparent moduli at 10% EL, 25% EL, 50% EL, and 75% EL for three diol dibenzoate candidates and DEHP. Standard deviation shown, *n* = 5. Abbreviations—DB: dibenzoate; EL: elongation.

	**Calculated apparent modulus (in MPa)**			
	At 10%EL	At 25%EL	At 50%EL	At 75%EL
1,3-propranediol DB	10.2 ±1.50	8.2 ± 0.36	5.5 ± 0.09	4.5 ± 0.21
1,5-pentanediol DB	9.6 ± 0.51	8.5 ± 0.28	6.1 ± 0.26	4.6 ± 0.23
1,6-hexanediol DB[a]	9.7 ± 0.50[a]	7.9 ± 0.48[a]	5.4 ± 0.21[a]	3.9 ± 0.12[a]
DEHP	22.1 ± 2.08	12.1 ± 0.57	6.8 ± 0.25	5.1 ± 0.22

[a] *n*=4

Table S2. Material property data collected for 40 phr PVC/1,4-BDB blends at room temperature, at least 48 h after processing. For $n \geq 3$, standard deviation is shown, and for $n = 2$ the experimental spread is shown.

Candidate plasticizer	**T_g by DSC (*n* = 3)**	**DMTA torsion (T) (*n* = 1)**		**Tensile Testing (at 20°C) (*n* = 8)**			**Hardness (at 20°C) (*n* = 2)**
	T_g (°C)	G' at 1Hz (MPa)	G" at 1Hz (MPa)	Elongation at break (%EL)	Max. stress (MPa)	Young's modulus (MPa)	Surface hardness (MPa)
1,4-butanediol DB	— / 2.5 ± 3.1[a]	1230 (30 °C) 5.1 (60 °C)	93 (30 °C) 1.3 (60 °C)	13.8 ± 6.7	19.8 ± 1.3	534 ± 29	18.8 ± 4.7

[a] Two separate results recorded for two heating cycles, separated by the slash: no T_g recorded on first heating cycle, second value represents data obtained in second heating cycle (see Figure 3A);

Table S3. Statistical analysis results. Software used: GraphPad Prism v.7.01 (La Jolla, CA, United States)

**ANOVA: Tg (five groups: 1,3-PrDB; 1,4-HDB; 1,5-PDB; 1,6-HDB; DEHP**			
**F value**	***p* value**	***p* value summary**	**Significance**
12.32	0.0007	***	Yes
**Bonferroni's multiple comparisons test**	**Significance**	**Summary**	**Adjusted p value**
1,3-PrDB vs. 1,4-BDB	No	ns	>0.9999
1,3-PrDB vs. 1,5-PDB	No	ns	0.0605
1,3-PrDB vs. 1,6-HDB	Yes	*	0.0161
1,3-PrDB vs. DEHP	Yes	**	0.0023
1,4-BDB vs. 1,5-PDB	No	ns	0.0945
1,4-BDB vs. 1,6-HDB	Yes	*	0.0246
1,4-BDB vs. DEHP	Yes	**	0.0034
1,5-PDB vs. 1,6-HDB	No	ns	>0.9999
1,5-PDB vs. DEHP	No	ns	0.6009
1,6-HDB vs. DEHP	No	ns	>0.9999
**All other tests from here on exclude 1,4-BDB, as it was statistically significantly different in all tests**			
**ANOVA: Elongation at break (four groups: 1,3-PrDB; 1,5-PDB; 1,6-HDB; DEHP)**			

**F value**	***p* value**	***p* value summary**	**Significance**
10.33	0.0009	***	Yes
**Bonferroni's multiple comparisons test**	**Significance**	**Summary**	**Adjusted p value**
1,3-PrDB vs. 1,5-PDB	Yes	**	0.0016
1,3-PrDB vs. 1,6-HDB	Yes	**	0.0071
1,3-PrDB vs. DEHP	Yes	**	0.0033
1,5-PDB vs. 1,6-HDB	No	ns	>0.9999
1,5-PDB vs. DEHP	No	ns	>0.9999
1,6-HDB vs. DEHP	No	ns	>0.9999
**ANOVA: Maximum recorded stress (four groups: 1,3-PrDB; 1,5-PDB; 1,6-HDB; DEHP)**			
**F value**	***p* value**	***p* value summary**	**Significance**
540.8	<0.0001	****	Yes
**Bonferroni's multiple comparisons test**	**Significance**	**Summary**	**Adjusted p value**
1,3-PrDB vs. 1,5-PDB	Yes	**	0.0037
1,3-PrDB vs. 1,6-HDB	Yes	***	0.0003
1,3-PrDB vs. DEHP	Yes	****	<0.0001
1,5-PDB vs. 1,6-HDB	No	ns	0.6721
1,5-PDB vs. DEHP	Yes	****	<0.0001
1,6-HDB vs. DEHP	Yes	****	<0.0001
**ANOVA: Apparent modulus at 25% EL (four groups: 1,3-PrDB; 1,5-PDB; 1,6-HDB; DEHP)**			
**F value**	***p* value**	***p* value summary**	**Significance**
98.58	<0.0001	****	Yes
**Bonferroni's multiple comparisons test**	**Significance**	**Summary**	**Adjusted p value**
1,3-PrDB vs. 1,5-PDB	No	ns	>0.9999
1,3-PrDB vs. 1,6-HDB	No	ns	>0.9999
1,3-PrDB vs. DEHP	Yes	****	<0.0001
1,5-PDB vs. 1,6-HDB	No	ns	0.2370
1,5-PDB vs. DEHP	Yes	****	<0.0001
1,6-HDB vs. DEHP	Yes	****	<0.0001
**ANOVA: Surface hardness (four groups: 1,3-PrDB; 1,5-PDB; 1,6-HDB; DEHP)**			
**F value**	***p* value**	***p* value summary**	**Significance**
25.98	<0.0001	****	Yes
**Bonferroni's multiple comparisons test**	**Significance**	**Summary**	**Adjusted p value**
1,3-PrDB vs. 1,5-PDB	No	ns	>0.9999
1,3-PrDB vs. 1,6-HDB	Yes	***	0.0002
1,3-PrDB vs. DEHP	No	ns	>0.9999
1,5-PDB vs. 1,6-HDB	Yes	***	0.0001
1,5-PDB vs. DEHP	No	ns	>0.9999
1,6-HDB vs. DEHP	Yes	***	0.0002

* ($p < 0.05$); ** ($p < 0.01$); *** ($p < 0.001$); **** ($p < 0.0001$)
